# VIM-1 carbapenemase-producing *Escherichia coli* isolated from retail seafood, Germany 2016

**DOI:** 10.2807/1560-7917.ES.2017.22.43.17-00032

**Published:** 2017-10-26

**Authors:** Nicole Roschanski, Sebastian Guenther, Thi Thu Tra Vu, Jennie Fischer, Torsten Semmler, Stephan Huehn, Thomas Alter, Uwe Roesler

**Affiliations:** 1 Freie Universitaet Berlin, Institute for Animal Hygiene and Environmental Health, Berlin, Germany; 2 Freie Universitaet Berlin, Institute of Food Safety and Food Hygiene, Berlin, Germany; 3 Federal Institute for Risk Assessment, Department Biological Safety, Berlin, Germany; 4 Robert Koch Institute, Berlin, Germany; 5 Beuth University of Applied Sciences, Life Science and Technology, Berlin, Germany

**Keywords:** Enterobacteriaceae, multidrug resistance, beta-lactamases

## Abstract

Carbapenems belong to the group of last resort antibiotics in human medicine. Therefore, the emergence of growing numbers of carbapenemase-producing bacteria in food-producing animals or the environment is worrying and an important concern for the public health sector. In the present study, a set of 45 Enterobacteriaceae isolated from German retail seafood (clams and shrimps), sampled in 2016, were investigated by real-time PCR for the presence of carbapenemase-producing bacteria. One *Escherichia coli* (ST10), isolated from a Venus clam (*Ruditapes philippinarum*) harvested in the Mediterranean Sea (Italy), contained the carbapenemase gene *bla*_VIM-1_ as part of the variable region of a class I integron. Whole-genome sequencing indicated that the integron was embedded in a Tn3-like transposon that also contained the fluoroquinolone resistance gene *qnr*S1. Additional resistance genes such as the extended-spectrum beta-lactamase *bla*_SHV-12_ and the AmpC gene *bla*_ACC-1_ were also present in this isolate. Except *bla*_ACC-1_, all resistance genes were located on an IncY plasmid. These results confirm previous observations that carbapenemase-producing bacteria have reached the food chain and are of increasing concern for public health.

## Introduction

In human medicine, carbapenems are one of the last treatment options for serious infections caused by multidrug-resistant Gram-negative bacteria [1]. Therefore, the increasing number of reports describing carbapenemase-producing Enterobacteriaceae are worrying. In the past six years, it has become obvious that the occurrence of carbapenemase-producing bacteria is no longer limited to clinical settings. Increasing numbers of carbapenemase-producing bacteria have been isolated from the environment, wild birds and companion and food-producing animals all over the world [2]. Although the use of carbapenems is prohibited in food-producing animals and restricted for pets in most European countries, these findings illustrate the continuous spread of these highly resistant bacteria accompanied by emerging public health problems. In 2011, the first VIM-1 producing *Salmonella* Infantis and *Escherichia coli* were isolated in German fattening farms for pigs and chickens [3,4]. 

European Union legislation implemented monitoring of carbapenem-resistance in *Salmonella* and *E. coli* in food-producing animals (chickens, turkeys, pigs and cattle) and the derived meat samples [5]. Similarly structured resistance surveillance programmes, targeting bacterial isolates derived from food-producing animals and retail meat, are in place globally [6]. Vegetables, fruits or seafood are frequently consumed raw and thus may become a source of antimicrobial resistant bacteria, including carbapenemase-producing microorganisms [6-8]. Microbial contamination of the environment with faecal bacteria is an important route of transmission. For example, bacteria in river water may move on to seas and oceans [9]. Therefore, seafood harvested from contaminated regions serves as a vehicle for the transmission of these bacteria [10]. On the other hand, aquaculture is a fast-growing food production sector [11]. 

To prevent bacterial infections in the farmed fish, intensive aquaculture is often accompanied by increased use of a wide range of chemotherapeutic agents, in particular antibiotics [11]. This situation supports the occurrence and spread of antibiotic-resistant bacteria in seafood products. Fish and seafood play an important role on the food market. The global food fish supply has increased at an average annual rate of 3.2% (1961–2013); fish consumption per capita increased similarly from an average of 9.9 kg/year in the 1960s to 20.1 kg/year in 2014 [12]. Several publications report the presence of antibiotic-resistant bacteria in seafood [10,13-15]. The first carbapenemase-producing bacteria derived from seafood were described in 2014, when a *bla*_VIM-2_ containing *Pseudomonas fluorescens* was isolated from a squid from South Korea [8]. One year later, a study described the occurrence of *bla*_OXA-48_-producing bacteria in 3.3% of the investigated seafood samples (squid, sea squirt, clams and seafood medley) from China and Korea [6].

In the present study, seafood samples from retail markets in Berlin, Germany, were investigated for the presence of carbapenemase-producing Enterobacteriaceae.

## Methods

### Detection of carbapenemase-producing Enterobacteriaceae

A set of 160 seafood samples (80 shrimp, 49 blue mussels, 15 Venus clams, 11 razor shells and five cockles) derived from 12 independent sellers in Berlin was sampled between December 2015 and August 2016 and initially investigated for the presence of ESBL/AmpC-producing bacteria (data not shown). In that set, 45 Enterobacteriaceae were isolated: *Klebsiella pneumoniae* (n = 13) and *E. coli* (n = 12) were the predominant species, followed by *Enterobacter cloacae* (n = 6), *Citrobacter freundii* (n = 5), *Hafnia alvei* (n = 3), *Pantoea septica* (n = 1), *Enterobacter aerogenes* (n = 1), *Morganella morganii* (n = 1), *Citrobacter braakii* (n = 1), *Enterobacter asburiae* (n = 1) and *Leclercia adecarboxylata* (n = 1). In the present study, the 45 DNA samples were additionally screened for the presence of the carbapenemase encoding genes *bla*_VIM_, *bla*_KPC_, *bla*_NDM_, *bla*_OXA-48_ and *bla*_GES_ by real-time PCR [16]. The detected *bla*_VIM-1_ gene was amplified and sequenced [17].

### Further characterisation of a *bla*_VIM-1_-containing *Escherichia coli* isolate

The phylogenetic group was determined by PCR [18], the class I integron was amplified and the purified amplification products were sequenced as described previously [4]. Plasmid DNA was isolated using the NucleoBond Xtra Midi kit (Macherey-Nagel, Dueren, Germany) and the *bla*_VIM-1_-containing plasmid was transferred into electrocompetent *E. coli* NEB10-beta (New England Biolabs, Frankfurt a.M., Germany). The incompatibility (Inc-) group of the plasmid was determined by using the PCR-based replicon typing (PBRT) kit (Diatheva, Fano, Italy). The size of the *bla*_VIM-1_-containing plasmid was estimated by S1 nuclease pulsed-field gel electrophoresis (PFGE) [19] using the following running conditions: 1–25 s, 17 h, 6 V/cm, 120 V. In addition, genomic DNA from the *E. coli* wild-type strain E-124–4 as well as the transformant T_E-124–4_ and the recipient strain NEB10-beta was isolated from overnight cultures of the selected isolates using the PureLink Genomic DNA Mini Kit (Invitrogen, Waltham, United States (US)). Sequencing libraries were generated with the Nextera XT DNA Sample Preparation Kit and paired-end sequencing was performed on the Illumina MiSeq benchtop using the MiSeq Reagent v3 600-cycle Kit (2 × 300 bp) (Illumina, San Diego, US). The raw data were assembled de novo using CLC Genomics workbench v.9.0 (http://www.clcbio.com/). Resistance genes, plasmid incompatibility groups as well as multilocus sequence types were identified using the Web-tools ResFinder 2.1 [20], PlasmidFinder 1.3 [21], VirulenceFinder 1.5 [22] and MLST 1.8 [23], using the scheme by Wirth et al. [24]. Plasmid sequences were extracted from the set of whole genome data by reductive sequence analysis of the transformant and the recipient strain. The resulting plasmid contigs where compared with available plasmid sequences using the Basic Local Alignment Search Tool (BLAST; National Center for Biotechnology Information; https://blast.ncbi.nlm.nih.gov/Blast.cgi?PAGE_TYPE=BlastSearch) and the software Geneious 7.1.2. Insertion sequence elements were detected by using the IS Finder (https://www-is.biotoul.fr/blast.php).

Minimal inhibitory concentrations for a set of different antibiotics were investigated using the VITEK-2 compact system and the AST-card N248 (bioMérieux, Nuertingen, Germany). In addition, a disc diffusion assay described by the norm CLSI-M02-A11 [25] was performed using the following carbapenem discs: meropenem (MEM) (10 µg), imipenem (IMI) (10µg) and ertapenem (ETP) (10 µg) (bestbion, Cologne, Germany). The obtained data were interpreted following Clinical and Laboratory Standards Institute (CLSI) guidelines CLSI-M100-S24 [26].

## Results

### Genotypic characterisation

Among the 45 investigated Enterobacteriaceae, one *E. coli* isolate (E-124–4) was found to be positive for the carbapenemase gene *bla*_VIM-1_. The isolate belonged to the phylogenetic group A, sequence type ST10, and was originally isolated from a Venus clam (*Ruditapes philippinarum*). Plasmid electroporation, replicon typing, S1 nuclease PFGE (data not shown) as well as whole genome sequence data (E-124–4: coverage 130×, contigs n = 157; T_E-124–4_: coverage 100×, contigs n = 203; NEB10-beta coverage 120×, contigs: n = 142) indicated that the carbapenemase gene was located on an IncY plasmid (pE-124–4) of ca 194 kb. Sequence analyses showed that *bla*_VIM-1_ was located within a class I integron (E-124-4 GenBank accession number: PDDP00000000). Besides the carbapenemase gene *bla*_VIM-1_, genes for the aminoglycoside 6'-N-acetyltransferase *aac*A4, the aminoglycoside phosphotransferase *aph*(3')-XV, the aminoglycoside adenylyltransferase *aad*A1 as well as the chloramphenicol acetyltransferase *cat*B2 were detected in the variable region of the integron (Figure).

**Figure:**
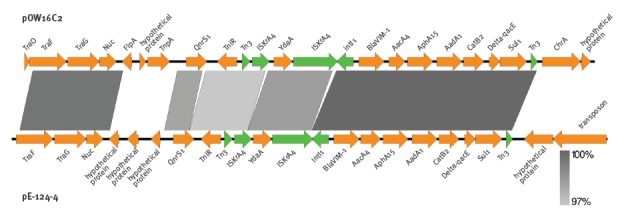
Comparison of the *bla*_VIM-1_-containing transposon region of the plasmids pOW16C2 isolated from *Klebsiella pneumoniae* derived from river water sampled in Switzerland and pE-124–4 isolated from *Escherichia coli* derived from retail seafood, Germany, 2016

GenBank accession number of the *bla*_VIM-1_ containing transposon region of pE-124-4: MG182343*.

In addition, the conserved region at the 3’- end contained the sulphonamide resistance gene *sul*1. Besides the class I integron, the investigated isolate harboured eight additional antibiotic resistance genes conferring resistance to different classes of antimicrobials (Table 1).

**Table 1:** Whole genome sequence comparison of the *Escherichia coli* wild-type isolate (E-124–4) derived from retail seafood and its transformant (T_E-124–4_), Germany, 2016

Isolate	ST	Resistance genes	Plasmid incompatibility group	Genes associated with virulence
E-124–4	ST-10	*aac*A4-like, *aad*A1, *aph*(3')-XV, ***bla*_ACC-1_**, *bla*_SHV-12_, *bla*_VIM-1_, *cat*B2, *dfr*A14-like, *mph*(A), *qnr*S1, *str*A-like, *str*B-like, *sul*1, *sul*2	IncY	*gad*
T_E-124–4_	ST-1060	*aac*A4-like, *aad*A1, *aph*(3')-XV, *bla*_SHV-12_, *bla*_VIM-1_, *cat*B2, *dfr*A14-like, *mph*(A), *qnr*S1, *str*A-like, *str*B-like, *sul*1, *sul*2	IncY	*gad, iss*
NEB10-beta	ST-1060	none	none	*gad, iss*

ST: sequence type.

E-124-4 GenBank accession number: PDDP00000000.

For comparison, the recipient strain NEB10-beta is also included. Except *bla*_ACC-1_ (bold letters), all of the detected resistance genes were transferred by electroporation.

The S1-PFGE (data not shown) confirmed the presence of a single plasmid in T_E-124–4_. A genome comparison of the wildtype as well as the transformant and the recipient strain showed that 13 of the 14 detected resistance genes were co-located on the *bla*_VIM-1_-containing IncY plasmid. Only the AmpC gene *bla*_ACC-1_ was not transferred to the recipient strain. The whole genome data indicated that this gene was located on the *E. coli* chromosome. At the 5’-end, the *bla*_ACC-1_ was associated with the insertion sequence IS*Ecp1* followed by the genes *sgb*E (2-ribulose-5-phosphate-4-epimerase), *rha*R (transcriptional activator) and *yic*J2 (inner membrane symporter) and on the 3’-end, IS*Kpn7* was detected adjacent to the genes *gdh*A (glutamate dehydrogenase), *ula*E (L-ribulose-5-phosphate-3-epimerase) and s*gb*H (3-keto-L-gluconate-6-phosphate-decarboxylase). Comparison of the extracted IncY plasmid sequences showed that the plasmid contained a mixture of different plasmid components and had low overall similarity with all published IncY plasmids carrying *bla*_VIM-1_. It is noteworthy that the class I integron detected here was located within a Tn3-like transposon, similar to the one previously reported in a *K. pneumoniae* isolated from a river in Switzerland [27]. The region located proximal to the class I integron contained the *qnr*S1 gene, *tni*R encoding a resolvase/integrase, the Tn3-like transposase, IS*KrA4* as well as the putative transposon resolvase *yda*A. However, at the distal end of the integron, only the Tn3-like transposase was located adjacent to the *sul*1 gene (Figure).

### Phenotypic characterisation

Besides the genetic background, we also investigated phenotypic resistances at two different time points: first, the minimal inhibitory concentration (MIC) for the wildtype isolate E-124–4 was determined immediately after the PCR-detection of the *bla*_VIM-1_ gene (11 August 2016). At this time point, no carbapenem resistance against IMI as well as MEM was detected using the VITEK-2 compact system (Table 2). Due to the fact that ETP was not included on the AST card N248 and the presence of the *bla*_VIM-1_ gene was already proved by sequencing, the results were reassessed by disc diffusion including the three carbapenems IMI, MEM and ETP. Thereby the following zone diameters were obtained: IMI: 21 mm; MEM: 24 mm; ETP: 25 mm. According to CLSI-M100-S24, these diameters indicated a carbapenem-sensitive phenotype. However, according to the European Food Safety Authority, the measured zone diameters of 24 mm for MEM and 25 mm for ETP were indicative of non-susceptibility to carbapenems [28]. A simultaneous cultivation in liquid medium containing either 2 µg/mL or 8 µg/mL meropenem indicated that the isolate was able to grow under selective conditions. 

**Table 2:** Minimum inhibitory concentrations of the *Escherichia coli* wild-type isolate (E-124–4) derived from retail seafood and its transformant (T_E-124–4_), Germany 2016

Isolate	Time of assessment	PIP	PIP-TAZ	CTX	CAZ	FEP	ATM	IMI	MEM	AMK	GE	TBM	CIP	TGC	FOS	COL	SXT
E-124–4	11 August 2016	≥ 128	≥ 128	4	≥ 64	≤ 1	16	≤ 0.25	≤ 0.25	≤ 2	≤ 1	2	≤ 0.25	≤ 0.5	≤ 16	≤ 0.5	≥ 320
E-124–4	21 September 2016	≥ 128	≥ 128	≥ 64	≥ 64	4	≥ 64	8	≥ 16	4	2	8	≤ 0.25	≤ 0.5	≤ 16	≤ 0.5	≥ 320
T_E-124–4_		≥ 128	f	≥ 64	≥ 64	≥ 64	≥ 64	8	8	8	2	8	0.5	≤ 0.5	≤ 16	2	≥ 320

AMK: amikacin; ATM: aztreonam; CAZ: ceftazidime; CIP: ciprofloxacin; COL: colistin; CTX: cefotaxime; f: failed run; FEP: cefepime; FOS: fosfomycin; GE: gentamicin; IMI: imipenem; MEM: meropenem; PIP: piperacillin; PIP-TAZ: piperacillin/tazobactam; TBM: tobramycin; TGC: tigecycline; SXT: trimethoprim/sulfamethoxazole.

Dark shading: resistant according to CLSI. Light shading: Intermediate according to CLSI.

Minimum inhibitory concentrations [µg/mL] determined by AST-card N248 (VITEK-2 compact system (bioMérieux, Nuertingen, Germany). The wild-type isolate (E-124–4) was investigated at two different time points (11 August 2016: after primary detection of the bla_VIM-1_ gene; 21 September 2016: after some weeks of cultivation in vitro). T_E-124–4_: transformant (NEB10-beta containing the IncY plasmid).

Following the completion of the other experiments such as plasmid preparation and transformation, the MIC determination was repeated on 21 September 2016 and showed that E-124–4 as well as its transformant T_E-124–4_ had gained full carbapenem resistance (Table 2).

## Discussion

Increasing numbers of antibiotic-resistant Enterobacteriaceae cause continuous problems in infection control and the global spread of carbapenemase-producing bacteria is especially worrisome [29]. In this study, we describe a *bla*_VIM-1_-containing *E. coli* (ST10) isolate derived from a Venus clam, harvested in the Mediterranean Sea (Italy) and purchased at a German retail market. This emphasises the importance of the food production chain in the global spread of antibiotic-resistant bacteria. 

At the same time, it has to be considered that the detection of carbapenemase producers is complex and that a single screening method is not always sufficient for the detection of this kind of isolate [30]. The first MIC determination using the AST-card N248 (bioMérieux) immediately after arrival of the samples suggested a carbapenem-sensitive phenotype according to CLSI-M100-S24. This was confirmed by a disc diffusion assay, as indicated by the CLSI guidelines. However, according to the European Food Safety Authority, the measured zone diameters for MEM as well as ETP were indicative of non-susceptibility to carbapenems [28]. When the wild-type isolate and its transformant were retested by MIC assay several weeks later, both of them exhibited a full resistant phenotype. The identification of bacterial isolates harbouring carbapenemase genes has frequently been described as challenging. Because the presence of a carbapenemase (e.g. KPC, VIM-1) not always leads to a high-level carbapenem resistance in laboratory testing, the isolates are often wrongly assessed as carbapenem-susceptible. Therefore, in human medicine, carbapenemase- producing bacteria are often detected only in case of therapy failure [31-34]. In 2015, Adams-Sapper et al. described that in the case of KPC-producing *K. pneumoniae,* a single exposure to a carbapenem was enough to generate subpopulations of high-level resistant bacteria [35]. Therefore, the real occurrence of carbapenem-resistant bacteria in food samples as well as food-producing animals or the environment can easily be underestimated. 

Moreover, it is important to mention the limitations of the present study. The screening was based on isolates initially selected on MacConkey agar plates containing 1 µg/mL cefotaxime. Therefore, isolates possessing a carbapenemase of the OXA family (e.g. OXA-48), without a coexisting resistance mechanism conferring resistance to oxyimino-cephalosporins such as extended spectrum- or AmpC-type beta-lactamases, may not have been detected. As the occurrence of carbapenemase-producing bacteria in effluent and sewage water, hospital sewage as well as river and coastal water samples has been described frequently, the pollution of seafood was merely a matter of time [36-40]. 

In addition, the detected *E. coli* sequence type ST10 is widespread among clinical as well as animal samples (e.g. seagulls) and wastewater [41-45]. In 2014 and 2015, a Canadian research group described the occurrence of *bla*_VIM-2_- and *bla*_OXA-48_-producing bacteria in seafood from China and Korea [6,8]. This study was followed by findings such as OXA-23-producing *Acinetobacter baumanii,* isolated from the fish *Pagellus acarne* harvested in the Mediterranean Sea [46], carbapenem-resistant *Enterobacter* derived from imported retail seafood in Canada [47] and VCC-1, a newly described Ambler class A carbapenemase from *Vibrio cholerae* isolated from imported retail shrimp sold in Canada [48]. These findings clearly demonstrate that carbapenemase-producing Enterobacteriaceae are present in the oceans and may enter the human food chain along this pathway. Our detection of a carbapenemase-producing *E. coli* isolated from a Venus clam, bought at a German retail market, supports this theory. 

This study showed that the *E. coli* isolate derived from a Venus clam possessed a mixture of different resistance genes and parts of resistance plasmids. Interestingly, a similar Tn3-like transposon, which harboured a class I integron consisting of the *bla*_VIM-1_ gene accompanied by *acc*A4, *aph* (3’)-XV, *aad*A1 and *cat*B2 in its variable region, was recently described in a *K. pneumoniae* isolated from a river in Switzerland [27]. However, the plasmid carrying this integron was different from the plasmid in our isolate. These findings underline the importance of mobile genetic elements (plasmids or transposons) for the spread of carbapenemase-producing bacteria and demonstrate that this is an important risk factor that deserves special attention. 

Because the clam was fished in Italy, we compared E-124–4 with previously published *bla*_VIM-1_ isolates reported from that country: the association of *bla*_VIM-1_ and *bla*_SHV-12_ on the same plasmid has already been described for Enterobacteriaceae derived from an Italian tertiary-care hospital [49], however, the detected plasmid replicon type was IncN, while the plasmid investigated here belonged to the IncY group. In addition, a similar class 1 integron was discovered in *Achromobacter xylosoxydans* isolated from a urine sample of an inpatient at the University Hospital of Verona, Italy [50]. Similarly to E-124–4, it contained *bla*_VIM-1_ accompanied by *acc*A4, *aph (3’)-XV* and *aad*A1 in its variable region. However, no *cat*B2 was detected in that *Achromobacter* isolate. Despite the integron similarity, no further consistencies among the two plasmids have been detected. While the plasmid pAX2220 was 30 kb in size and did not contain an additional *bla*_SHV-12_, pE-124–4 was much larger, ca 194 kb. 

Moreover, it has to be taken into account that Venus clams are also served as a raw appetiser and that seafood is preferred raw in some regions, providing ideal conditions for the transmission and spread of the carbapenemase-producing bacteria or a transfer of the respective plasmids.

 This situation emphasises the importance of further monitoring programmes as well as the need for the seafood samples to be included into the national surveillance programmes. Similarly, comprehensive intervention strategies focussing on the prudent use of antibiotics as well as the prevention of an environmental spread of the resistant bacteria are crucial in human as well as veterinary medicine.

## *Author’s correction

The GenBank accession number of the blaVIM-1 containing transposon region of pE-124-4 was originally published as MG18234. This number was corrected to MG182343 on 9 November 2017.
