# Author’s correction for Euro Surveill. 2017;22(43)

**DOI:** 10.2807/1560-7917.ES.2017.22.45.171139c1

**Published:** 2017-11-09

**Authors:** Eurosurveillance editorial team

**Affiliations:** 1 European Centre for Disease Prevention and Control (ECDC), Stockholm, Sweden

In the article ‘VIM-1 carbapenemase-producing Escherichia coli isolated from retail seafood, Germany 2016’, published on 26 October 2017, the GenBank accession number of the blaVIM-1 containing transposon region of pE-124-4 was erroneously indicated as MG18234. The correct number is MG182343. On request of the authors, the manuscript was updated accordingly on 9 November 2017.

